# Optimization of Overdriving Pulse for Luminance Stability of Electrowetting Displays

**DOI:** 10.3390/mi16101085

**Published:** 2025-09-25

**Authors:** Yanjun Yang, Zichuan Yi, Wanzhen Xu, Jiashuai Wang, Qingsong Lu, Qifu Liu, Liming Liu, Feng Chi

**Affiliations:** 1 School of Electronic Information, University of Electronic Science and Technology of China, Zhongshan Institute, Zhongshan 528402, China; 2024024416@m.scnu.edu.cn (Y.Y.); 2024024402@m.scnu.edu.cn (Q.L.); l13137360827@163.com (Q.L.); liulmxps@126.com (L.L.); chifeng@semi.ac.cn (F.C.); 2 Guangdong Provincial Key Laboratory of Optical Information Materials and Technology, South China Academy of Advanced Optoelectronics, South China Normal University, Guangzhou 510006, China; 2024010350@m.scnu.edu.cn; 3 School of Integrated Circuit Science and Engineering (Exemplary School of Microelectronics), University of Electronic Science and Technology of China, Chengdu 611731, China; 202511012418@std.uestc.edu.cn

**Keywords:** electrowetting display (EWD), overdriving pulse, electrowetting principle, linear conversion, Luminance stability

## Abstract

As a reflective display technology, electrowetting displays (EWDs) have the advantages of a paper-like appearance, fast response speed, and full-color capability. However, the use of an overdriving voltage to improve the response speed of EWDs can cause fluctuations in display luminance, which manifest as glitches in the luminance change curve. In order to eliminate this luminance instability phenomenon, a new driving pulse is proposed, which consists of an overdriving phase, a switching phase, and a driving phase. Firstly, a simplified equivalent circuit model is proposed to apply a target voltage in the driving phase without break down of the hydrophobic insulating layer. Secondly, a COMSOL (Version 6.3) two-dimensional model is established to simulate the oil contraction process and conduct comparisons, so as to ensure the effectiveness of the overdriving pulse. Then, the overdriving phase is applied to improve oil response speed, and a linear function is used in the switching phase to alleviate glitch phenomena. Moreover, the influences of overdriving voltage, overdriving time, and linear switching time on the luminance curve are analyzed by charge trapping theory in order to obtain optimal performance. The experimental results show that the glitch phenomenon is eliminated effectively, and the luminance of the EWD is increased by 1.02% and 1.96% compared with the step switching pulse and PWM pulse, respectively, while the response time is shortened by 1.82% and 8.05% compared with the step switching pulse and PWM pulse, respectively.

## 1. Introduction

Electrowetting display (EWDs) [1], as emerging reflective display technologies, have become a research hotspot in the field of display technology due to their significant advantages, such as high reflectivity [2], fast response speed, and low power consumption [3,4]. However, although EWD technology has many potential advantages, it still faces many challenges in terms of practical application, such as luminance reduction caused by charge trapping [5]. Therefore, it is of great significance to solve these problems by optimizing the overdriving pulse in order to promote the commercialization and industrialization of EWD technology. So far, most studies on EWDs mainly aim to optimize or design a new driving pulse to reduce oil splitting [6,7,8], oil backflow [9,10,11], charge trapping [12,13,14], or optimize pixel structure [15,16,17], aiming to improve performance metrics such as aperture ratio, response speed, and luminance stability. Among them, response speed is an important indicator. To improve the response speed of droplets or pixels, researchers have proposed a variety of gradually optimized driving strategies and methods. Early research mainly focused on enhancing response performance by applying an overdriving voltage. For example, by studying the spreading behavior and response time of droplets under different DC and overdriving conditions, appropriate overdriving signals were selected to improve the response speed of viscous fluids [18]. Similarly, regarding the issue of response time for the rising use of electrowetting-driven low-viscosity liquid columns, studies have found that, compared with DC driving, the overdriving pulse can effectively reduce the response time [19]. Meanwhile, to further shorten the response time of the devices, an input voltage function with multiple exponential rise times was adjusted [20]. With the deepening of research, single-voltage control gradually evolved into multi-stage pulse design to balance speed and stability. For instance, a driving pulse based on the overdriving voltage and charge trapping theory was proposed, which shortens the response time of EWDs while taking charge trapping control into account [21]. To suppress oil backflow in the active matrix, a separated reset pulse was designed. In the first stage, an overdriving voltage is used to increase the response speed, while the voltage of all pixels is inverted to achieve a stable state [22]. Aiming at the impact of the hysteresis effect, an equivalent driving pulse design method with an overdriving voltage was proposed, which effectively improved the response performance [23]. Based on the analysis of the oil backflow phenomenon, a new driving pulse using an exponential function was proposed. This pulse increases the voltage exponentially, starting from the threshold voltage, and combines with overvoltage driving to suppress splitting and improve response speed [24]. To further break through performance bottlenecks, research has begun to focus on both pixel structure and pulse. At the pixel structure level, a multi-electrode pixel structure was proposed. This multi-electrode pixel structure divides the pixel into four square sub-electrodes, and a three-dimensional EWD simulation model was established for verification, improving the response speed through physical design [25]. At the pulse design level, to suppress charge trapping and mitigate the impact of leakage current, an efficient driving pulse was proposed. It uses an overdriving voltage in the driving stage to accelerate the response speed and maintains the target voltage in the stable stage to sustain the state [26]. Similarly, a multi-channel DC overdriving pulse was proposed, which divides the pulse into a start-up driving stage and a stable driving stage. The introduction of an overdriving voltage in the start-up stage significantly improved the response speed and enhanced grayscale consistency [27]. In addition, a new composite function pulse based on a sampling function has been proposed in recent years, which consists of an initial driving stage and a backflow suppression stage. In the initial driving stage, an overdriving voltage is used to quickly drive the pixel, and the overdriving voltage is attenuated to the target voltage, which not only improves the response speed but also effectively prevents damage to the pixel [28]. It can be seen that the above studies improve the response speed in different ways, most of which involve adding to the overdriving voltage to improve the response speed. However, when using overdriving voltage, the phenomenon of glitches in the luminance curve is ignored, which affects the stability of the luminance of the display.

To eliminate the glitch phenomenon in the luminance curve caused by overdriving voltage, a new pulse is proposed in this paper based on overdriving pulse and the electrowetting principle. Then, a COMSOL two-dimensional model is established to verify the feasibility of the designed pulse, and the relationship between overdriving voltage and overdriving time is studied. Lastly, a linear switching phase is designed to alleviate abrupt voltage changes and improve the luminance stability of the EWD.

## 2. Theory

### 2.1. Principle of EWD

**Figure 1:**
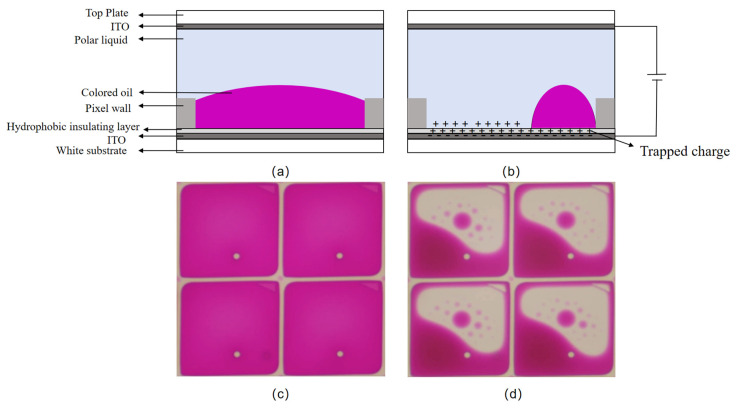
Structure of EWD pixels. (**a**) EWD in the “off” state; (**b**) EWD in the “on” state; (**c**) physical drawing of EWD in “off” state; (**d**) physical drawing of EWD in “on” state.

The pixel structure of EWDs mainly consists of the top glass, ITO electrode, pixel wall, polar liquid, colored oil, hydrophobic insulating layer (fluoropolymer), and bottom substrate. When no voltage is applied, the oil covers the whole bottom of the pixel, the pixel presents the color of the oil at this time, and the EWD is in the “off” state, as shown in Figure 1a,c. On the contrary, when a certain voltage is applied between the electrodes, the surface tension of the liquid changes and the liquid is pushed or diffused, so that the surface of the hydrophobic insulating layer can be displayed, as shown in Figure 1b,d.

In order to analyze the relationship between driving voltage and oil movement, and given that the capacitance of the pixel wall can be neglected due to the low conductivity of the photoresist material, a simplified equivalent circuit model [29] is proposed. The current in the hydrophobic insulating layer and oil film obeys Ohm’s law, so that the effective capacitance and effective current of a single pixel can be obtained, as shown in Equations (1) and (2).

(1) $$C=C_{oil}+C_{D}=\varepsilon_{0}(\frac{\varepsilon_{oil}S_{oil}}{h}+\frac{\varepsilon_{D}S_{pixel}}{d})$$

(2) $$I_{C}=C\frac{dV}{dt}$$

where C and $I_{C}$ are, respectively, the effective capacitance and effective current of a single pixel; $C_{oil}$ and $C_{D}$ are, respectively, the capacitances of the oil and the hydrophobic insulating layer; $\varepsilon_{0}$ is the vacuum dielectric constant; $\varepsilon_{oil}$ and $\varepsilon_{D}$ are, respectively, the dielectric constants of the oil and the hydrophobic insulating layer, $S_{oil}$ is the area where oil shrinks to the corners of a pixel; and $S_{pixel}$ is the area of a single pixel. h and d are, respectively, the thicknesses of the oil and the hydrophobic insulating layer, and V is the applied driving voltage. In addition, the correspondence between contact angle and driving voltage can be described by the Lippmann–Young equation [30], which can be modified according to refs. [30,31], as shown in Equation (3).

(3) $$\cos\theta =1-\frac{CV^{2}}{2\gamma }$$

where θ is the contact angle and γ is the interfacial tension between the colored oil and the conductive solution. It can be seen that the contact angle is proportional to the driving voltage. At the same time, the relationship between the thickness of the hydrophobic insulating layer and the driving voltage can be obtained from Equations (1) and (3), as shown in Equation (4).

(4) $$V=\sqrt{\frac{2(1-\cos\theta )\gamma }{C}}∼\sqrt{d}$$

**Figure 2:**
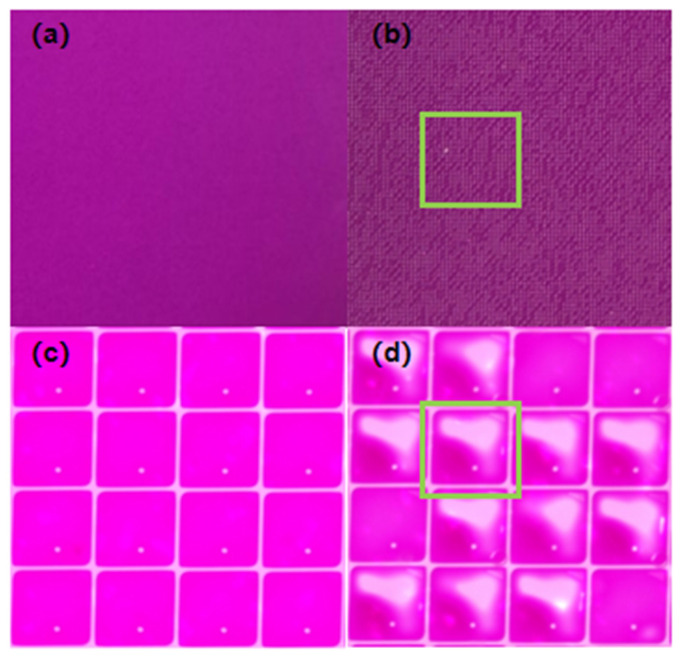
(**a**) An undamaged EWD under visual inspection; (**b**) a damaged EWD under visual inspection; (**c**) the pixel structure of the undamaged EWD under a microscope; (**d**) the pixels of the damaged EWD under a microscope.

It can be seen that the driving voltage is proportional to the thickness of the hydrophobic insulating layer. When the driving voltage increases to a critical value, a phenomenon occurs where the contact angle no longer decreases as the voltage continues to rise, which is known as contact angle saturation [30]. Subsequent further increases in the driving voltage will disrupt the stability of the oil, leading to the failure of the EWD. When no voltage is applied, the oil in the pixel should spread evenly across the bottom. This phenomenon, as observed with the naked eye and under a microscope, is shown in Figure 2a,c, respectively. However, after the EWD fails, colored oil droplets remain trapped in the corners of the pixel and cannot spread back or even overflow into adjacent pixels, forming scattered white spots. This phenomenon, as observed with the naked eye and under a microscope, after failure is shown in Figure 2b,d, respectively, where the regions of obvious failure are highlighted with green frames. Therefore, an excessively high driving voltage should not be applied during the experiment.

In addition, when a driving voltage is applied between the electrodes of the EWD, ions in the polar liquid are pulled toward the hydrophobic insulating layer by an electric field. These ions may enter the layer and become trapped, leading to charge accumulation. At this time, a reverse electric field is generated, as shown in Equation (5) and Figure 1b.

(5) $$V_{T}=\frac{Q_{T}}{C_{D}}$$

where $V_{T}$ is the electric potential generated by charge trapping and $Q_{T}$ is the amount of trapped charge. Equation (3) is modified to include the effect of charge trapping [31], as shown in Equation (6).

(6) $$\cos\theta =1-\frac{C{\left(V-V_{T}\right)}^{2}}{2\gamma }$$

It can be seen that as the drive voltage increases the influence of charge trapping becomes greater.

### 2.2. EWD Simulation Model

#### 2.2.1. Governing Equation

To investigate the dynamic response characteristics of the oil interface and the generation mechanism of the glitch phenomenon under the action of the overdriving voltage from the perspective of fluid dynamics, the phase-field method was used to study the dynamic process of the two-phase flow interface [32,33,34,35]. The governing equation of this model is shown in Equation (7).

(7) $$\begin{array}{l} \frac{\partial \varphi }{\partial t}+u\cdot \nabla \varphi =\nabla \cdot \frac{\gamma \lambda }{\varepsilon_{pf}^{2}}\nabla \psi \\ \psi =-\nabla \cdot \varepsilon_{pf}^{2}\nabla \varphi +(\varphi^{2}-1)\varphi +\frac{\varepsilon_{pf}^{2}}{\lambda }\cdot \frac{\partial f}{\partial \varphi }\\ \lambda =\frac{3\varepsilon_{pf}}{\sqrt{8}} \end{array}$$

where u is the velocity of the fluid, γ is the mobility parameter, ∇ is the gradient operator, $\varepsilon_{pf}$ is the capillary width, and λ is the energy density; both φ and ψ are phase-field variables, where φ is used to distinguish and track two immiscible fluids, with φ=1 representing oil and φ=−1 representing water. In this model, the motion of the liquid on the solid surface is indirectly tracked by solving the variables ψ and φ in the equation [36,37]. Also, to describe the dynamic motion of the immiscible two-phase flow, the transfer of mass and momentum is governed by the incompressible Navier–Stokes equations [38], as shown in Equations (8) and (9).

(8) $$\rho \left(\frac{\partial u}{\partial t}+u\cdot \nabla u\right)=-\nabla p+\nabla \cdot \left(\mu \left(\nabla u+{\left(\nabla u\right)}^{T}\right)-\frac{2}{3}u\left(\nabla \cdot u\right)I\right)+F$$

(9) $$F=F_{st}+\rho g+F_{vf}$$

where p represents the pressure of the fluid, ρ represents the density of the fluid, μ represents the dynamic viscosity of the fluid, F represents the external force, I is the unit matrix, and each term in Equation (8) represents inertial force, pressure, viscous force, and external force, respectively. External force consists of surface tension, gravity, and volume force, where $F_{st}$, ρg, and $F_{vf}$ represent surface tension, gravity, and volume force, respectively. In addition, in this multi-physics field coupled model, the laminar flow field is coupled to the electrostatic field by applying volume forces to the Navier–Stokes equations, and the electrostatic field can be obtained by calculating the divergence of the Maxwell stress tensor [32]. In the establishment of the two-dimensional EWD simulation model, the Maxwell stress tensor is shown in Equation (10).

(10) $$T=\left[\begin{array}{cc} T_{xx} & T_{xy}\\ T_{yx} & T_{yy} \end{array}\right]$$

Since the laminar flow change caused by the electrostatic field is reflected by the volume force, the expression of the volume force can be obtained as shown in Equation (11).

(11) $$F=\left[\begin{array}{cc} \frac{\partial \left(T_{xx}\right)}{\partial x} & \frac{\partial \left(T_{xy}\right)}{\partial y}\\ \frac{\partial \left(T_{yx}\right)}{\partial x} & \frac{\partial \left(T_{yy}\right)}{\partial y} \end{array}\right]$$

#### 2.2.2. Boundary Conditions

In this multi-physics field coupling model, it is necessary to determine the corresponding boundary conditions to obtain the solution for the governing equations. For the boundary conditions of the electrostatic field, the top of the model is grounded, the bottom outer boundary is connected to the designed pulse, and the remaining boundaries are assigned to zero charge. For laminar boundary conditions, the liquid–solid interface is defined as a wet wall condition, the initial values of pressure and velocity in the laminar field are set to 0, and the wall condition is set to Navier slip. At the same time, the interface of two-phase flow is selected as the initial boundary condition, and both sides of the model (except the pixel wall) are selected as the exit conditions.

#### 2.2.3. Model Simulation

**Figure 3:**
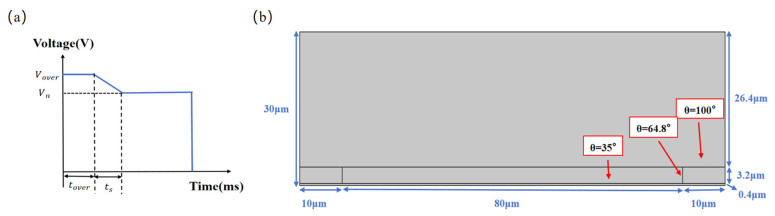

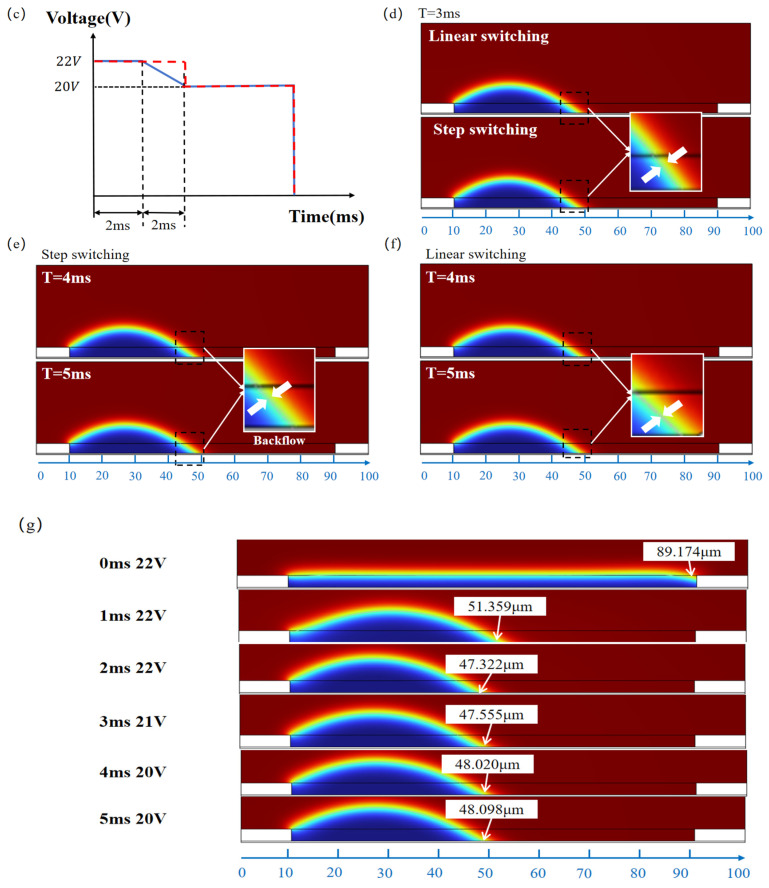
The 2D geometric model and simulation comparison. (**a**) The designed pulse, where $t_{s}$ represents the switching phase time, $V_{n}$ represents the stable driving voltage value, and $V_{\mathrm{over}}$ and $t_{\mathrm{over}}$ represent the overdrive voltage and time, respectively; (**b**) the 2D geometric model; (**c**) a comparison of step and linear switching pulse; (**d**) the oil contraction behavior under different pulses at 3 ms; (**e**) the oil contraction behavior under the step switching pulse at 4 ms and 5 ms; (**f**) the oil contraction behavior under the linear switching pulse at 4 ms and 5 ms; (**g**) simulation of oil contraction under the proposed linear driving pulse from 0 ms to 5 ms.

A two-dimensional simulation model was established, based on Table 1, and its model is shown in Figure 3b. The water and oil in the model were set to incompressible flow. Assuming that the temperature of the fluid remains constant, the thermal expansion of the fluid can be ignored and the effect of pressure on dynamic viscosity can be ignored.

Based on the aforementioned model, simulation verification was conducted for the designed pulse. As shown in Figure 3a, this pulse incorporates a direct linear function between the overdriving voltage and the target voltage, which can effectively mitigate abrupt voltage changes and achieve the effect of eliminating glitches. In addition, in order to verify whether the designed pulse in this paper had the effect of eliminating glitches, model simulation verification and comparison were carried out first. The designed linear switching pulse and step switching pulse were introduced into the designed simulation model, as shown in Figure 3c. In the figure, the solid blue line represents the designed pulse, and the dashed red line represents the step switching pulse. Both the designed pulse and step switching pulse have an overdriving voltage of 22 V and a target voltage of 20 V. Additionally, the overdriving time and switching time of the designed pulse are both set to 2 ms, and the overdriving time of the step pulse is set to 4 ms. Within the first 2 ms, the oil shrinks rapidly to one side of the model. Then, with the increase in time, since the voltage of the step switching model remains constant, the oil contraction state remains unchanged, while the driving voltage of the designed linear switching model decreases linearly, resulting in an increase in contact angle so that the oil has a backflow phenomenon, but this backflow phenomenon is not obvious. Figure 3d shows the oil contraction behavior under different pulse modes at 3 ms. The region between the white arrows shows the differences in oil contraction state between the step and linear switching pulses, which arise from their different applied voltage strategies. With the step switching pulse, the voltage remains constant, and the oil contraction state remains essentially unchanged; in contrast, under the linear switching pulse, the voltage decreases linearly over time, causing the slight backflow of the oil. In Figure 3e, the region between the white arrows shows that, at 4 ms and 5 ms, the step switching pulse induces significant backflow due to the voltage abrupt change, which manifests as a sharp glitch on the luminance curve. In contrast, the region between the white arrows in Figure 3f indicates that under the linear switching pulse, the voltage decreases linearly, resulting in a much gentler oil recession process; when the voltage reaches the target value, the oil contraction state stabilizes, thereby effectively suppressing glitches in the luminance curve. Figure 3g shows the dynamic response of the simulation model within the critical time range of 0 ms to 5 ms after the application of the linear pulse voltage. During the overdrive phase (0–2 ms), the relatively high driving voltage causes the oil film to contract rapidly. Subsequently, during the linear switching phase (2–4 ms), as the driving voltage decreases smoothly, the oil–water interface undergoes a smooth and continuous contraction under the combined influence of surface tension and the electric field force. No significant backflow is observed, indicating that the dynamic behavior during this phase is effectively controlled. Finally, during the steady voltage driving phase (4–5 ms), the oil stabilizes near the target position. Therefore, the simulation results show that the designed pulse can effectively eliminate the glitch phenomena in the luminance curve, verifying its effectiveness in the theoretical model. To further confirm the practical performance of this pulse, an experimental platform was built for experimental verification and is detailed in the next section.

## 3. Experimental Results and Discussion

### 3.1. Experimental Platform

In order to test the validity of the pulse, an experimental platform [39,40] was developed. The platform consisted of an input system and a test system. The input system consisted of a computer, a waveform generator for inputting the pulse, and a signal amplifier for amplifying the pulse signal. The test system consisted of a computer and a colorimeter for testing the response time of EWD.

An EWD was used as the test object in this experiment. The manufacturing process parameters of the EWD are shown in Table 2. In the test process, the pulse was designed and compiled in Matlab (Version R2024A) on the computer, and then the pulse was edited by the Arbexpress waveform editing software (Version 3.4). Then, the edited pulse was imported into the function generator through a universal serial bus (USB) interface, amplified by the signal amplifier, and measured by a colorimeter. Finally, the luminance data was recorded in real time by Admesy software (Version 2.9.3).

### 3.2. Pulse Design

**Figure 4:**
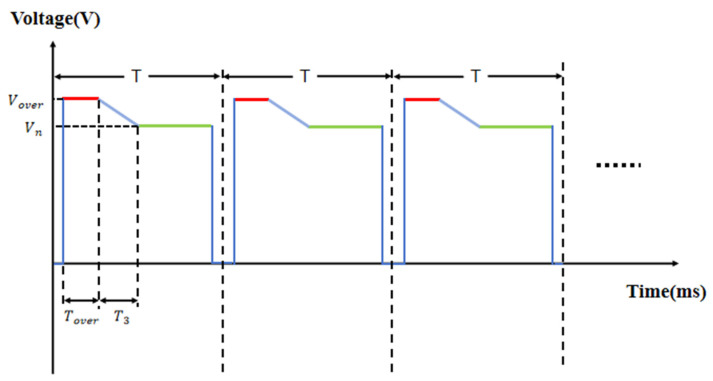
The proposed EWD driving pulse, which consists of an overdriving phase (bold red section), a switching phase (bold blue section), and a driving phase (bold green section), where $V_{\mathrm{over}}$ and $T_{\mathrm{over}}$ represent the overdriving voltage and the overdriving time, respectively, $T_{3}$ represents the switching time, and $V_{n}$ represents the steady driving voltage.

Figure 4 shows the designed driving pulse for mitigating glitch phenomena. The proposed pulse consists of an overdriving phase, a switching phase, and a driving phase. In the overdriving phase, an overdriving voltage is applied to improve the response speed of the oil, and this duration is called the overdriving time. In the switching phase, the linear function is designed to prevent abrupt voltage changes and abrupt luminance changes, so as to optimize the phenomenon of luminance instability. During the driving phase, a target voltage of 20 V was applied to ensure that the EWD was not damaged during the experiment. Charge trapping affects both the response time and luminance of the EWD, and linear switching can slow down capacitive discharge to eliminate glitches. Therefore, it is necessary to experimentally determine the overdriving voltage, overdriving time, and linear switching time to achieve optimal performance.

### 3.3. Overdriving Phase Testing

**Figure 5:**
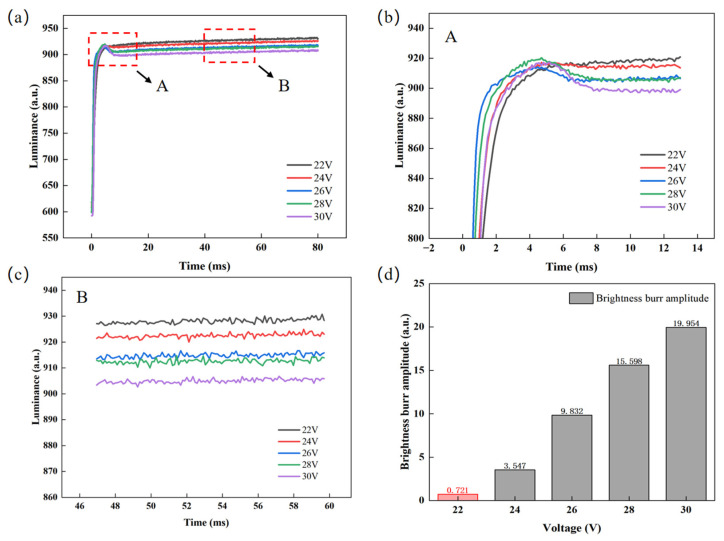
Test data for different overdriving voltages. (**a**) Luminance–time curves at different overdriving voltages, where the response times corresponding to overdriving voltages of 22 V, 24 V, 26 V, 28 V, and 30 V are calculated to be 65.55 ms, 66.93 ms, 68.27 ms, 69.34 ms, and 71.29 ms, respectively; (**b**) enlarged view of area A in Figure 5a; (**c**) enlarged view of area B in Figure 5a; (**d**) comparison of luminance glitch amplitudes under different overdriving voltages.

For the designed pulse, this experiment initially sets the switching phase duration to 2 ms and uses a colorimeter to measure the luminance rise curve of the EWD. This approach was adopted to analyze the effects of overdriving voltage and duration on luminance response characteristics and glitch phenomena. When analyzing the effects of overdriving voltage, overdriving voltages were set to 22 V, 24 V, 26 V, 28 V, and 30 V, with a constant duration of 4 ms, so as to prevent experimental errors caused by structural damage to partial pixels under excessively high voltage [30]. The luminance curves of different overdriving voltages are shown in Figure 5a. It can be seen that when the applied voltage is lower than the threshold voltage of 10 V, the EWD is in the “off state”, corresponding to a luminance level of 210. Once the applied voltage exceeds 10 V, the oil begins to retract. The response time is defined as the time interval from the initial moment until the luminance reaches its average steady-state level, where the average steady-state luminance is calculated as the arithmetic mean of the last 30 data points in each voltage column. Meanwhile, the difference between the maximum luminance value when the glitch phenomenon occurs and the minimum luminance value it drops to afterward is defined as the glitch amplitude. When the glitch amplitude is less than 2, it can be regarded as normal luminance curve fluctuation. The experimental results show that as the overdriving voltage increases, the rising speed of luminance first accelerates and then slows down. When the voltage remains below 26 V, raising the voltage accelerates the response; however, once the voltage exceeds 26 V, the rising speed decreases. The reason for this phenomenon is that when the overdriving voltage is higher than 26 V, it drives more ions to migrate to the hydrophobic insulating layer and induce charge trapping. These trapped charges form a reverse electric potential, leading to a decrease in the effective driving voltage. At the same time, when the overdriving voltage is suddenly switched to the target voltage, the charges stored in the capacitor will be rapidly discharged through the circuit, causing the luminance to decrease. This sudden change forces the oil to shrink rapidly, the luminance rises briefly and then decreases as the electric field weakens, forming glitch phenomena. It can be seen from Figure 5b,d that when the overdriving voltage is 22 V the curve is smooth, without obvious fluctuation, and the glitch amplitude is the smallest; when the overdriving voltage is greater than or equal to 24 V, the glitch amplitude of luminance is more obvious. In addition, it can be observed from Figure 5c that as the overdriving voltage increases, the stabilized luminance value decreases. Therefore, increasing the overdriving voltage will lead to an increase in glitch amplitude, an extension of response time, and a decrease in stabilized luminance. When the overdriving voltage is 22 V, the response time is the shortest, 65.55 ms, the luminance is the highest, 930.51, and the glitch amplitude is the lowest, 0.721. Therefore, the overdriving voltage was set to 22 V for a better display effect.

**Figure 6:**
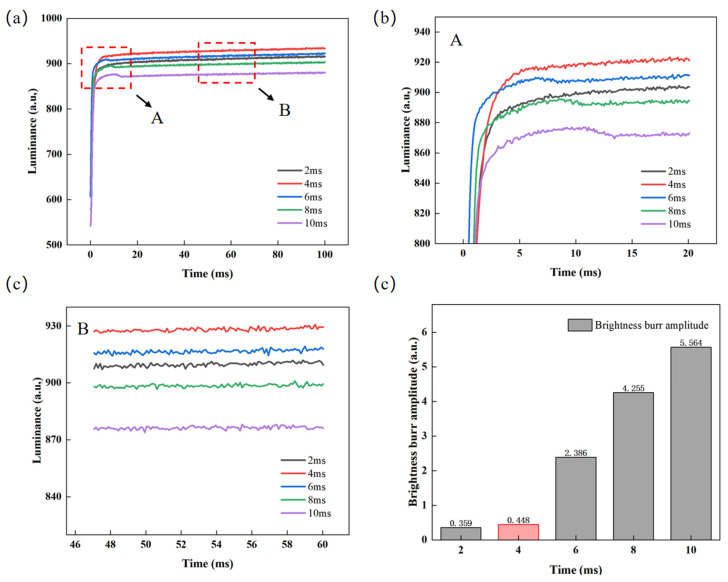
Test data for different overdriving times. (**a**) Luminance–time curves for different overdriving times, where the response times corresponding to overdriving times of 2 ms, 4 ms, 6 ms, 8 ms, and 10 ms are calculated to be 67.5 ms, 65.55 ms, 67 ms, 68.5 ms, and 70.12 ms, respectively; (**b**) enlarged view of area A in Figure 6a; (**c**) enlarged view of area B in Figure 6a; (**d**) luminance glitch amplitude contrast diagram at different overdriving times.

After determining the optimal parameters of the overdriving voltage, the influence of the overdriving time on luminance and the glitch phenomenon is further analyzed by testing the luminance–time curve. The overdriving voltage was set to 22 V, and the overdriving time was set to 2 ms, 4 ms, 6 ms, 8 ms, and 10 ms, respectively. The luminance rise curves corresponding to different overdriving times are shown in Figure 6a. At first, with the increase in overdriving time, the luminance rises faster and faster, and the luminance increases gradually. However, when the overdriving time increases to a certain value and continues to increase, the luminance rise speed decreases and glitches appear, as shown in Figure 6b. The reason for this phenomenon is that the excessive overdriving time (>6 ms) increases the time maintaining the reverse electric field, resulting in more accumulated charges in the hydrophobic insulating layer, reducing the effective driving voltage greatly. Meanwhile, the stabilized luminance decreases and the glitch amplitude increases, as shown in Figure 6d. Meanwhile, as shown in Figure 6c, the glitch amplitude is approximately 0 when the overdriving time is 2 ms or 4 ms, and the luminance value is the highest at 930.51 when the overdriving time is 4 ms. Therefore, the overdriving time was set to 4 ms for a better display effect.

### 3.4. Test for Switching Phases

**Figure 7:**
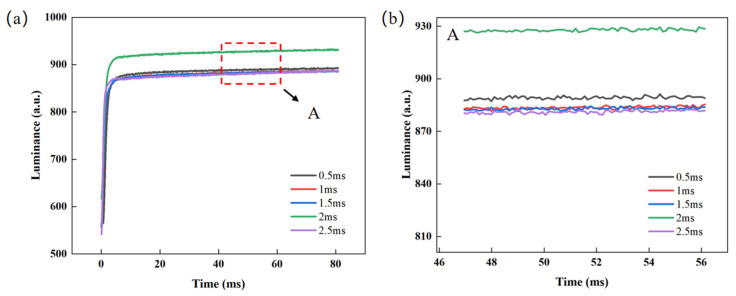
Test data for different switching times. (**a**) Luminance–time curves at different switching times, where the response times corresponding to switching times of 0.5 ms, 1 ms, 1.5 ms, 2 ms, and 2.5 ms are calculated to be 66.2 ms, 65.8 ms, 66 ms, 65.55 ms, and 66.5 ms, respectively; (**b**) enlarged view of area A in Figure 7a.

In the test experiments for the overdriving phase, it is concluded that when the overdriving voltage is 22 V and the overdriving time is 4 ms, the glitch amplitude value of the luminance curve is already less than 2, which can be regarded as normal fluctuation. To further improve display performance metrics such as response speed and luminance, the timing of the linear switching phase was investigated. This phase uses linear function switching to eliminate the glitch phenomenon. The voltage and time of the overdriving phase were set to 22 V and 4 ms, respectively, and the switching phase time was set to 0.5 ms, 1 ms, 1.5 ms, 2 ms, and 2.5 ms, respectively, for testing. The luminance rise curves under different switching times are shown in Figure 7a. It is observed that the average glitch amplitude in the luminance curve remains below 2, while the response time shows almost no change as the switching time increases. As the switching time increases, the slope of the linear function decreases gradually, leading to a reduction in switching speed. In Figure 7b, when the switching time is less than 2 ms, the luminance slightly decreases as the switching time increases. This is primarily because longer switching times lead to increased charge trapping, accompanied by capacitive discharge effects. However, the linearly decreasing voltage effectively mitigates abrupt voltage changes, thereby suppressing the rapid discharge of the capacitor, resulting in minimal changes in luminance. When the switching time is greater than 2 ms, the response time is extended, and the luminance level is relatively low. Meanwhile, when the switching time is 2 ms, the highest luminance is obtained, which is 930.51. Therefore, the switching time was set to 2 ms.

### 3.5. Performance Comparison

**Figure 8:**
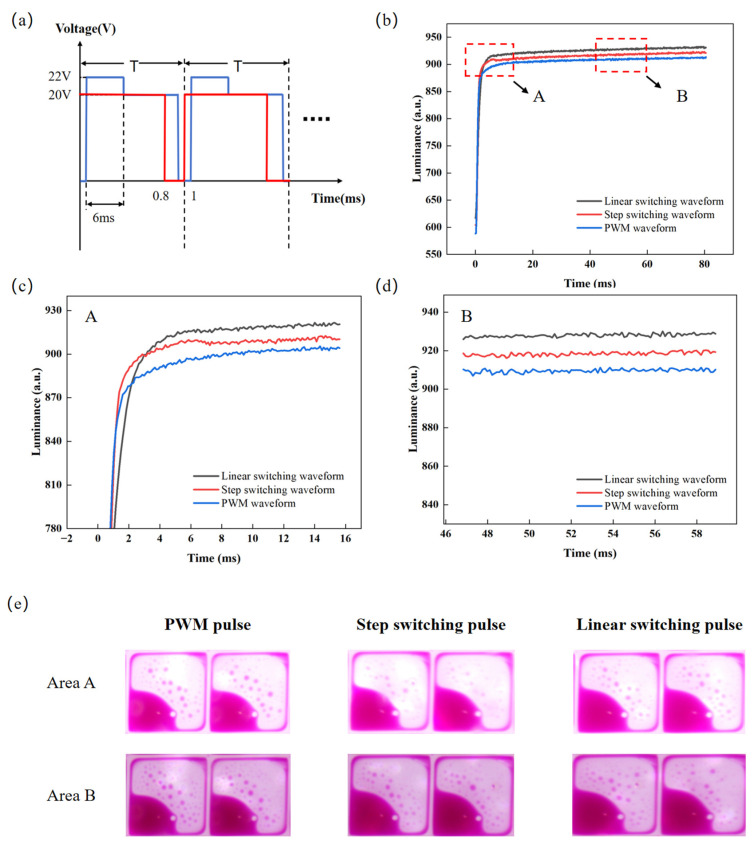
A comparison among the linear switching pulse, step switching pulse, and PWM pulse. (**a**) PWM pulse and step switching pulse. (**b**) Luminance–time curves under the proposed pulse, step switching pulse, and PWM pulse. (**c**) Enlarged view of area A in Figure (**b**). (**d**) Enlarged view of area B in Figure (**b**). (**e**) Photographs of the instantaneous displays of the EWD states corresponding to the two areas in Figure 8b: (top row) the display state during the glitch occurrence phase (area A); (bottom row) the display state during the stable phase (area B). The corresponding driving pulses are as follows: (left) PWM pulse, (middle) step switching pulse, (right) linear switching pulse proposed in this paper.

According to the experimental results, the overdriving voltage, the overdriving time, and the linear switching time of the designed pulse are set to 22 V, 4 ms, and 2 ms, respectively. In order to prove the effect of the designed pulse, it was compared to the PWM pulse [41,42] and the step switching pulse [21]; the PWM pulse and the step switching pulse are shown in Figure 8a. The voltage amplitude of the PWM pulse is 20 V, and the duty ratio is 80%. The overdriving voltage and the overdriving time of the step switching pulse are 22 V and 6 ms, respectively, and the target voltage is 22 V. The luminance rise curves of the proposed pulse, the PWM pulse, and the step switching pulse are shown in Figure 8b. It can be seen that the response time of the proposed pulse is shorter than that of the step switching pulse, the luminance is higher, and the glitch amplitude is effectively reduced. Specifically, as can be seen from Figure 8c, the glitch amplitude of the proposed pulse is less than 2, while the glitch amplitude of the step switching pulse is 2.347. This indicates that the proposed pulse effectively suppresses the luminance glitches caused by the overdriving voltage. Meanwhile, the response time of the proposed pulse is 65.55 ms, the response time of the step switching pulse is 66.77 ms, and the response time of the PWM pulse is 71.29 ms. Therefore, the response time is shortened by 1.82% compared with the step switching pulse and by 8.05% compared with the PWM pulse. The luminance curves of the proposed pulse, the PWM pulse, and the step switching pulse after their input stabilizes are shown in Figure 8d. The steady luminance of the proposed pulse is 930.51, the steady luminance of the step switching pulse is 921.04, and the steady luminance of the PWM pulse is 912.65. Compared with the step switching pulse and the PWM pulse, the achievable luminance is increased by 1.02% and 1.96%, respectively. Figure 8e and Table 3 show the photographs of the instantaneous display states at the selected positions during the glitch occurrence phase (area A) and the stable phase (area B), along with their corresponding aperture ratio values. The PWM pulse and the proposed pulse maintain a constant aperture ratio in both the glitch occurrence phase and the stable phase, while the step switching pulse has a larger aperture ratio in the glitch occurrence phase than in the stable phase. It can be observed that the proposed pulse achieves a uniform and stable transition from the overdriving voltage to the driving voltage, and compared with the two reference driving methods, it realizes higher luminance and superior stability in the stable phase. In EWDs, the step switching of the overdriving voltage causes an abrupt change in the electric field, leading to the rapid discharge of the equivalent capacitor and the generation of a reverse electric field, thereby inducing glitch phenomenon. In this study, by optimizing the overdriving pulse, transient changes in the electric field are mitigated, achieving the goal of eliminating glitches. Meanwhile, compared with the step switching pulse and the PWM pulse, the proposed pulse improves both luminance and response speed.

## 4. Conclusions

Aiming at the glitch phenomenon in EWDs under overdriving voltages, a multi-stage driving pulse strategy is proposed. By integrating three stages, namely overdriving, switching, and driving, this method effectively ensures the reliability of the device while improving the response performance. Meanwhile, combined with electrowetting theory and equivalent circuit modeling, a quantitative relationship between the driving voltage and the thickness of the insulating layer is established, which provides a basis for the safe selection of the target voltage. Moreover, the results of multi-physics simulation show that changing traditional step switching to linear gradient control can significantly suppress oil backflow and sudden changes in the electric field. This linear transition mechanism effectively delays the capacitive discharge process and reduces the accumulation of charge trapping effects, thereby inhibiting the formation of reverse electric fields and significantly reducing luminance fluctuations. The proposed driving strategy not only realizes glitch-free display but also significantly improves optical response speed and stability, providing a reliable solution for the design of driving systems for high-performance EWDs.

## Figures and Tables

**Table 1:** EWD 2D simulation model parameters.

Parameters	Quantity	Value	Unit
Material	Density of oil	763	kg/m^3^
	Density of water	998	kg/m^3^
	Dynamic viscosity of oil	2 × 10^−3^	Pa·s
	Dynamic viscosity of water	1.01 × 10^−3^	Pa·s
	Dielectric constant of oil	4	1
	Dielectric constant of water	80	1
	Dielectric constant of hydrophobic insulating layer	1.934	1
	Dielectric constant of pixel wall	3.28	1
Structure	Width of pixel	100	μm
	Height of pixel wall	3.2	μm
	Width of pixel wall	10	μm
	Thickness of hydrophobic insulating layer	0.4	μm
	Thickness of oil	3.2	μm
Interfacial	Surface tension of oil	0.02	N/m
	Contact angle at top of pixel wall	100	deg
	Contact angle on side of pixel wall	64.8	deg
	Contact angle of hydrophobic insulating layer	35	deg

**Table 2:** EWD manufacturing process parameters.

Substrate	Hydrophobic Insulating Layer Thickness	FP Curing	Activation	Pixel Wall Height	Oil Color	Descum	Backflow
0.55 mm UP ITO	400 nm	185 °C 30 min	Power 10 W Time 6 s	3.5 μm	Magenta (R21 5%)	Power 100 W Time 100 s	200 °C/1 h

**Table 3:** The aperture ratios in different regions corresponding to different pulses.

	PWM Pulse	Step Switching Pulse	Linear Switching Pulse
**The aperture ratio of area A (%).**	60.42	62.25	61.48
**The aperture ratio of area B (%).**	60.39	60.07	61.45

## Data Availability

Data are contained within the article.

## Abbreviation

The following abbreviation is used in this manuscript:

EWD	Electrowetting display
